# The Pathways from a Behavior Change Communication Intervention to Infant and Young Child Feeding in Bangladesh Are Mediated and Potentiated by Maternal Self-Efficacy

**DOI:** 10.1093/jn/nxx048

**Published:** 2018-02-27

**Authors:** Amanda A Zongrone, Purnima Menon, Gretel H Pelto, Jean-Pierre Habicht, Kathleen M Rasmussen, Mark A Constas, Francoise Vermeylen, Adiba Khaled, Kuntal K Saha, Rebecca J Stoltzfus

**Affiliations:** 1 Poverty, Health, and Nutrition Division, International Food Policy Research Institute, Washington, DC; 2 Division of Nutritional Sciences, Charles H Dyson School of Applied Economics and Management, and Cornell Statistical Consulting Unit, Cornell University, Ithaca, NY; 3 Division of Charles H Dyson School of Applied Economics and Management, and Cornell Statistical Consulting Unit, Cornell University, Ithaca, NY; 4 Division of Cornell Statistical Consulting Unit, Cornell University, Ithaca, NY; 5 Department of Nutrition for Health and Development, WHO, Geneva, Switzerland

**Keywords:** self-efficacy, maternal self-efficacy, potentiation, mediation, complementary feeding, infant and young child feeding, Bangladesh, behavior change communication, behavior change intervention, randomized controlled trial

## Abstract

**Background:**

Although self-efficacy is a potential determinant of feeding and care behaviors, there is limited empirical analysis of the role of maternal self-efficacy in low- and middle-income countries. In the context of behavior change interventions (BCIs) addressing complementary feeding (CF), it is possible that maternal self-efficacy can mediate or enhance intervention impacts.

**Objective:**

In the context of a BCI in Bangladesh, we studied the role of maternal self-efficacy for CF (MSE-CF) for 2 CF behaviors with the use of a theoretically grounded empirical model of determinants to illustrate the potential roles of MSE-CF.

**Methods:**

We developed and tested a locally relevant scale for MSE-CF and included it in a survey (*n* = 457 mothers of children aged 6–24 mo) conducted as part of a cluster-randomized evaluation. Qualitative research was used to inform the selection of 2 intervention-targeted behaviors: feeding green leafy vegetables in the last 24 h (GLV) and on-time introduction of egg (EGG) between 6 and 8 mo of age. We then examined direct, mediated, and potentiated paths of MSE-CF in relation to the impacts of the BCI on these behaviors with the use of regression and structural equation modeling.

**Results:**

GLV and EGG were higher in the intensive group than in the nonintensive control group (16.0 percentage points for GLV; *P* < 0.001; 11.2 percentage points for EGG; *P* = 0.037). For GLV, MSE-CF mediated (β = 0.345, *P* = 0.010) and potentiated (β = 0.390, *P* = 0.038) the effect of the intensive group. In contrast, MSE-CF did not mediate or potentiate the effect of the intervention on EGG.

**Conclusions:**

MSE-CF was a significant mediator and potentiator for GLV but not for EGG. The divergent findings highlight the complex determinants of individual specific infant and young child feeding behaviors. The study shows the value of measuring behavioral determinants, such as MSE-CF, that affect a caregiver's capability to adopt intervention-targeted behaviors.

## Introduction

Adequate and appropriate complementary feeding (CF) with nutrient-dense foods such as green leafy vegetables and eggs beginning at 6 mo of age is important for child survival, growth, and development (1, 2). In low- and middle-income countries, including Bangladesh, inadequate CF during the period from 6 to 24 mo is associated with stunting and other manifestations of undernutrition (1, 3–8). Consequently, numerous interventions to improve CF have been designed and implemented, including behavior change communication (BCC), sometimes coupled with food provision or cash transfers to caregivers (9–11).

Designing effective BCC for CF requires understanding the key factors and processes that influence caregiver behavior. Several well-developed theoretical models of behavior change [e.g., the Health Belief Model (12), the Theory of Planned Behavior (13, 14), and Social Cognitive Theory (15, 16)] have been developed to inform the design and evaluation of nutrition interventions (17). A central construct of these theories is self-efficacy, which Bandura (18, 19) defines as “beliefs in one's capabilities to organize and execute the courses of action required to produce given levels of attainments” (18; p. 624). Bandura asserts that self-efficacy is domain-specific and that measures of generalized self-efficacy are less analytically useful when examining its role in behavior and behavior change, because an individual's self-efficacy varies across activities and functions (18).

Maternal self-efficacy has long been considered a key determinant of breastfeeding success. Work in this area has included the development of validated self-efficacy scales and applications in low- and high-income countries (20). Such work has been lacking for CF, although a small body of empirical research has documented the importance of self-efficacy as a driver of behavior change in nutrition interventions in low- and middle-income countries (21–23). Previously, self-efficacy has been examined empirically as either a mediator (21) or as a potentiator (i.e., positive effect modifier) (22) of nutrition behavior change interventions (BCIs). However, to our knowledge, no research has examined both roles for self-efficacy, even though theory suggests that self-efficacy can play out in multiple, and highly specific, ways (18). In addition, little is known about how self-efficacy is related to behavior change for CF practices, because behavioral theories are rarely considered when designing or evaluating such programming (24). Given these gaps, we sought to examine the role of maternal self-efficacy for CF (MSE-CF) in the context of an intervention that considered issues of maternal self-efficacy both in the design and planning of the intervention. Methodologic rigor was accomplished by combining the inferential strengths afforded by experimental design with contextualized understanding generated by qualitative methods.

Alive & Thrive (A&T) is a global initiative that aims to improve infant and young child feeding (IYCF) practices in multiple contexts (25, 26). The A&T behavior change program theory explicitly recognized the importance of self-efficacy in the intervention pathway and thus targeted improved maternal self-efficacy along with other behavioral determinants, such as knowledge, skills, intent, motivation, and perceived social norms (25, 26). The impact evaluation of the initiative showed that a combination of strategies—interpersonal counseling, mass media, and community mobilization (the “intensive intervention” or intervention group)—led to greater improvements in all core WHO CF indicators than did mass media alone (the “nonintensive intervention” or control group) (26). However, impacts varied by behavior, suggesting that multiple factors likely influenced the effect of the intervention on specific behaviors (26).

In this substudy, a component of the overall evaluation, we used cross-sectional midline survey data collected 2 y after the baseline survey in Bangladesh. With the explicit goal of examining issues of MSE-CF, this survey included specific measures of MSE-CF that were extensively tested to increase measurement precision. We aimed to assess the extent to which the increased intake of particular intervention-targeted foods was mediated by increased MSE-CF and whether MSE-CF multiplied the intervention's effect, thereby acting as a “potentiator” or positive effect modifier. Put simply, we asked: Did the intervention act through increasing MSE-CF? And did mothers with higher MSE-CF gain more from the intervention than mothers with lower MSE-CF?

## Methods

### Intervention and experimental design

Mothers living in the intensive intervention areas received age-specific, targeted, and frequent interpersonal counseling on IYCF practices from frontline health workers. In addition, their communities were targeted with social mobilization to build awareness of IYCF practices among men and other family members. In contrast, mothers in the nonintensive group received standard interpersonal counseling on nutrition from frontline health workers in the context of routine health visits, along with less-structured community mobilization that did not cover IYCF practices (26). A mass media campaign on IYCF was implemented nationwide for the duration of the intervention (25–27).

For the primary impact evaluation of A&T, 20 rural subdistricts in Bangladesh were randomized to receive the 2 arms of BCC interventions described above: intensive or nonintensive. The overall evaluation included multiple waves of data collection, with primary impact findings reported from a baseline survey in 2010 and an endline survey in 2014 (27). The details of the intervention (25, 26) and the evaluation (26, 27) are described elsewhere.

### Research questions

We used 2 food-specific behaviors promoted by the intervention—namely, routine feeding of green leafy vegetables (proxied with the use of a 24-h recall) to children aged 6–24 mo and on-time introduction of eggs between 6 and 8 mo of age—to test the following 3 hypotheses:

1. Intervention effects: More mothers reported feeding green leafy vegetables in the last 24 h and introducing eggs on time to infants and young children in the intensive group than in the nonintensive group.
2. Mediating role of MSE-CF: MSE-CF will be higher in the intensive group than in the nonintensive group, and in the intensive group, MSE-CF will be positively associated with reported practice of both the 2 promoted behaviors.
3. Potentiating role of MSE-CF: More mothers with higher MSE-CF in the intensive group would reportedly practice both behaviors, compared with mothers in the intensive group with lower MSE-CF.

### Data source and study sample for the MSE-CF– focused analysis

We developed and tested a specific measure of MSE-CF (described below) and then integrated these measures into a midline cross-sectional survey conducted in July 2012 in 10 randomly selected subdistricts among the 20 evaluation subdistricts (5 subdistricts in each arm) (26). Survey respondents (mothers) answered questions about feeding behaviors for a child between 6 and 24 mo of age. If the mother had >1 child between 6 and 24 mo of age, the youngest child in this age range was selected as the index child. The survey included a total of 457 mothers, of which 213 were in the intensive areas and 244 were in the nonintensive areas. This study was approved by the institutional review boards at the International Food Policy Research Institute and Cornell University. The overall impact evaluation, the results of which are reported elsewhere (26), was approved by the Bangladesh Medical Research Council.

### Measures and variables

#### Creation of MSE-CF questions and scale

A set of questions related specifically to MSE-CF were developed on the basis of self-efficacy theory (28) and the specific CF behaviors prioritized in the intervention (25). Attention was given to designing questions that supported the principles of appropriate scale development (29). The questions were subjected to cognitive testing (30) to determine if the question constructs, terminology, and translation were appropriate for respondents. In this development and testing phase, native Bengali-speaking interviewers conducted individual qualitative interviews with 15 mothers who were not in the survey sample. They asked respondents to “think aloud” as the question was asked, systematically going through each part of the question (i.e., lead-in, terminology, response scale) and determining how well the translation reflected the original English questions. Mothers were most easily able to answer questions where the response was scaled by first answering “yes,” “no,” or “don't know” to the lead-in question; “yes” answers were then followed up by asking “always” or “sometimes.” This procedure generated a 3-point response scale: “yes—always,” “yes—sometimes,” or “no.” The cognitive testing revealed that, for these concepts, this approach worked better than a standard 5-point Likert scale.

The MSE-CF scale was composed of 4 questions (informed by this cognitive testing) on the following items: maternal self-efficacy for *1*) feeding family-cooked foods, *2*) avoiding feeding store-bought snacks, *3*) being able to decide the types of foods fed to the child, and *4*) raising a healthy child. The responses to these 4 questions were added to generate an MSE-CF scale with a range from 0 to 8. A “no” response was given a value of 0, “yes—sometimes” a value of 1, and “yes—always” a value of 2. None of the respondents replied “don't know.” The scale had an internal consistency Cronbach's α (29) coefficient of 0.60 in the total study sample. MSE-CF was centered at the group mean for all analysis.

#### Selection of outcome variables

Early process evaluation research highlighted the likelihood that challenges to the adoption of the intervention-promoted behaviors would be behavior-specific (31). Additional qualitative research conducted as part of the larger process evaluation (32) and A&T's prioritization of interventions informed our selection of the 2 outcome variables. We chose 2 behaviors to address several characteristics:

1. The behaviors were specifically targeted by the intervention.
2. The behaviors had contrasting attributes in terms of the mother's control over them, ease of implementation, and potential cultural and economic constraints.
3. The behaviors reflected contrasting cultural classification as a “family food” compared with a “special food” for the child.

##### Outcome variable—consumption of green leafy vegetables in the last 24 h

In the intensive areas, mothers were instructed by frontline health workers to give infants and young children green leafy vegetables alone or mashed with thick lentils, other vegetables, and either fish or egg into rice. Green leafy vegetables are commonly consumed as part of the daily diet in rural Bangladesh (33) and can be grown in or around the homestead; therefore, they were easily available to mothers. In the midline survey, mothers were asked: “Did your child eat green leafy vegetables yesterday (during the day or night)?” A binary variable was constructed from the yes or no responses to this question.

##### Outcome variable—the on-time introduction of egg

Eggs were chosen because of their positioning as a special food for children. Although eggs were less expensive than other animal-source foods, they often were not served as a family food because fish is a preferred animal-source food among Bangladeshi adults. This positions egg, in contrast to green leafy vegetables, as a potential “special food” that is less accessible to mothers than are green leafy vegetables.

The intervention encouraged an earlier age of introduction of egg (specifically between 6 and 7 mo) than is common in Bangladesh, where animal-source foods are often introduced late (34). To examine response to this recommended practice we asked: “At what age did you start giving eggs to [index child's name]?” Mothers had the option of responding, “not yet given.” Mothers who reported giving their child egg between 6 and 8 mo of age were coded as “on time.” All responses of giving their child egg at ≥9 mo old were considered “not on time.”

#### Covariate variables

We chose covariates to include in regression and structural equation models on the basis of previous research (25, 31, 35) on potential influences in the Bangladeshi context on the 2 behavioral outcomes. These variables were as follows: child sex, child age (only in the green leafy vegetable models), maternal education and socioeconomic status, household food security, and consumption of green leafy vegetables or egg by any household members apart from the index child in the last 24 h. Maternal education was calculated on the basis of the highest level of schooling, reported by the mother. Socioeconomic status was measured with the use of an index of 32 assets, including both durable assets and livestock assets (36). Household food insecurity was assessed with the use of the Household Food Insecurity Access Scale (37).

Household consumption of green leafy vegetables or egg in the last 24 h was included in the regression models as a proxy for the household availability of either green leafy vegetables or egg. Given previous research in this context on the association between maternal consumption and child consumption, we presumed that household availability would influence whether these foods were given to the child (33). The intervention did not deliver messages that encouraged all household members to consume these foods, and at the time of this midline survey the intervention had no detectable effect on household consumption of these foods. Thus, the intervention would have affected child consumption of these foods through 1 of 2 pathways: increased feeding of an available family food to the child or special provisioning of a food not consumed by the family for the child. Because neither pathway affects household consumption of these foods, adjusting for household consumption is appropriate and does not “over control.”

### Statistical analysis

We used a modified intent-to-treat analysis that was based on a single-difference, partial sample of household-level cross-sectional survey data, accounting for the clustered randomization. The intent-to-treat analysis makes the conservative assumption that, in the context of this large-scale BCI, the intervention effects are represented by the differences between those living in intensive areas and those in nonintensive areas.

#### MSE-CF as a mediator

To guide our initial analysis of MSE-CF as a mediator, we used a 4-step test for mediation with the use of logistic and ordinary least-squares (OLS) regression (38–40).

##### Step 1: Establishing intervention effects

We examined intervention effects on the 2 outcome variables, the feeding of green leafy vegetables in the last 24 h and on-time introduction of egg.

##### Step 2: Intervention associations with MSE-CF

We examined intervention effects on MSE-CF.

##### Step 3: MSE-CF's association with outcome variables

We examined the association between the MSE-CF scale score and the feeding of green leafy vegetables in the last 24 h or on-time introduction of egg, without including the A&T intervention variable.

##### Step 4: Evidence for mediation

We tested mediation with the use of a full model that included feeding of green leafy vegetables in the last 24 h or on-time introduction of egg as the dependent variable and also included the A&T intervention variable and MSE-CF score as independent variables.

#### MSE-CF as a potentiator

To examine potentiation by MSE-CF, we tested for an interaction between the MSE-CF scale and A&T intervention variable in the multiple regression models.

#### MSE-CF as a simultaneous mediator and potentiator

Finally, we combined the regressions described above into 2 models, one for feeding of green leafy vegetables in the last 24 h and one for on-time introduction of egg, with the use of structural equation modeling. Structural equation modeling allowed us to simultaneously model both the indirect and direct effects of the A&T intervention on the outcome variables as well as the interaction term, while correctly estimating SEs. With the use of this model, we generated a β-coefficient with a corrected SE for the mediation term.

#### Adjusting models and statistical software

All of the regressions presented in this section were conducted adjusting for all sociodemographic control variables (except MSE-CF, where appropriate) along with clustering. Predicted probabilities were calculated to improve the interpretability of the results for all of the logistic regression analyses.

All of the analysis used STATA 13 (StataCorp). For OLS regression we used the *reg* command, for logistic regression we used the *logit* command, and to generate predicted probabilities we used the *margins* command. For structural equation modeling we used the *gsem* command, followed by the *nlcom* command to generate the mediation term. To adjust for clustering, we used the *cluster* command. Data were removed through list-wise deletion when there were missing data for a variable included in the model.

## Results

### Sample characteristics

In our sample, the 463 children were, on average, 14 mo old and 51% female. Mothers were ∼26 y old and had completed 5 y of schooling. The Household Food Insecurity Access Scale score indicated that these families had low levels of food insecurity. These sample characteristics were distributed evenly between the intensive and nonintensive groups (**Table 1**) and were comparable to the baseline values in the overall evaluation sample (**Supplemental Table 1**).

**TABLE 1:** Characteristics of the sample of children aged 6–24 mo and descriptive statistics on dependent, independent, and control variables^1^

Intervention group	Intensive (*n* = 217)	Nonintensive (*n* = 246)
Age of child, mo	14.0 ± 5.23	13.6 ± 5.25
Sex of child, % female	50.2	52.0
Mother's age, y	26.0 ± 5.18	25.5 ± 5.88
Maternal education, schooling completed, y	5.48 ± 3.36	5.00 ± 3.25
SES, all-assets score	12.8 ± 3.72**	11.9 ± 3.61
Household food insecurity,^2^ HFIAS score	1.59 ± 3.21	1.83 ± 3.66
MSE-CF score	6.92 ± 1.25****	6.14 ± 1.69
Young children consuming green leafy vegetables in the last 24 h, %	41.0****	23.2
Young children with on-time introduction of egg (age 6–8 mo), %	78.4** [199]	66.7 [213]
Age of introduction of egg, mo	7.30 ± 2.46** [199]	8.13 ± 2.99 [213]
Households consuming green leafy vegetables in the last 24 h, %	45.6	44.7
Households consuming egg in the last 24 h, %	22.6	24.0

^1^Values are means ± SDs unless otherwise indicated; *n* in brackets (*n* values are not shown when missing values are <5%). Unadjusted *P* values: *P < 0.05; **P < 0.01; ***P < 0.001; ****P < 0.0001. HFIAS, Household Food Insecurity Access Scale; MSE-CF, maternal self-efficacy for complementary feeding; SES, socioeconomic status.

^2^The range of possible scores for HFIAS is 0–27, with lower scores reflecting better food security.

The mean ± SD MSE-CF score was 6.50 ± 1.55 (range: 1–8). The MSE-CF score was higher in the intensive group (6.92 ± 1.25) than in the nonintensive group (6.14 ± 1.69) (*P* < 0.0001, unadjusted). More mothers fed green leafy vegetables to their children in the last 24 h in the intensive group than in the nonintensive group: 41.0% compared with 23.2% respectively (*P* < 0.0001, unadjusted). The percentage of children with on-time introduction of egg was significantly greater in the intensive group (78.4%) than in the nonintensive group (66.7%) (*P* = 0.0079, unadjusted). There were no significant differences between groups in household consumption of green leafy vegetables or egg in the last 24 h (Table 1).

### Green leafy vegetables

#### Intervention effects

In a fully adjusted model, before introducing MSE-CF, mothers in the intensive group were more likely to feed green leafy vegetables in the last 24 h (1.01 increase in the log odds; *P* < 0.001; results not shown). These log odds correspond to a 16.0 percentage-point increase in the predicted probability of feeding green leafy vegetables in the last 24 h between the intensive (39.8% predicted probability) and nonintensive (23.8% predicted probability) groups.

#### Mediation effects

Mothers in the intensive group had a higher MSE-CF score than did those in the nonintensive group in fully adjusted OLS regression (β = 0.766, *P* = 0.001; results not shown). With the use of logistic regression, we also found a significant association between the MSE-CF scale and the feeding of green leafy vegetables in the last 24 h (β = 0.273, *P* = 0.003; results not shown).

When we included MSE-CF in the full model, we found that the β-coefficient for the intensive group was attenuated but still significant (0.874 increase in the log odds, *P* = 0.001; results not shown). This translates to a 13.7 percentage-point increase in the predicted probability of the feeding of green leafy vegetables in the last 24 h between the intensive and nonintensive groups (25.0% predicted probability in the nonintensive group and 38.7% predicted probability in the intensive group). The MSE-CF scale was significant in the full model for green leafy vegetables (*P* = 0.018; results not shown). We thus found that MSE-CF partially mediated between being in the intensive group and the feeding of green leafy vegetables in the last 24 h. These results do not support full mediation because the intervention variable was still significant when MSE-CF was controlled for.

#### Potentiation by MSE-CF

When including the multiplicative interaction term between the intervention and MSE-CF in the logistic regression model, we found a 0.395 (*P* = 0.031) increase in the log odds of feeding green leafy vegetables in the last 24 h with each 1-unit increase in the MSE-CF scale (results not shown).

#### Simultaneous mediation and potentiation by MSE-CF

**FIGURE 1:**
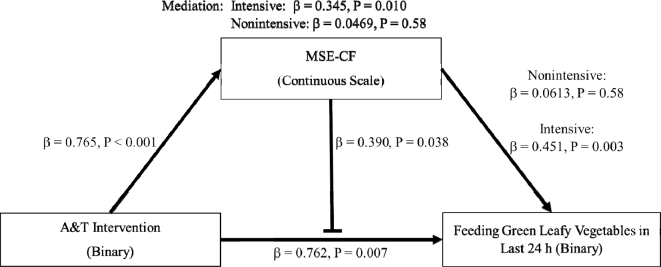
Fully adjusted structural equation model for the partial mediation through MSE-CF in the intensive group and the simultaneous potentiation of MSE-CF for the feeding of green leafy vegetables in the last 24 h (*n* = 457). A&T, Alive & Thrive; MSE-CF, maternal self-efficacy for complementary feeding.

**FIGURE 2:**
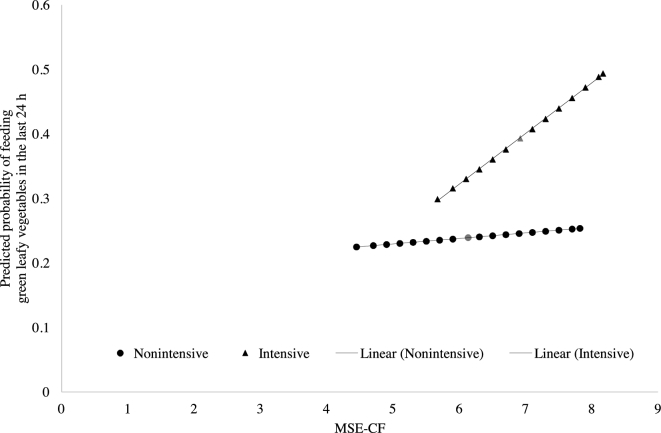
A graphic depiction of the structural equation model for the partial mediation through MSE-CF in the intensive group and the simultaneous potentiation of MSE-CF for the feeding of green leafy vegetables in the last 24 h (*n* = 457). MSE-CF, maternal self-efficacy for complementary feeding.

In the fully adjusted structural equation model (**Figure 1**), being in the intensive group was associated with a 0.765-point increase in MSE-CF (*P* < 0.001) and a 0.762 increase in the log odds of feeding green leafy vegetables in the last 24 h (*P* = 0.007), when compared with the nonintensive group. A 1-unit increase in MSE-CF was associated with a 0.451 increase in the log odds of feeding green leafy vegetables in the last 24 h in the intensive group (*P* = 0.003). In contrast, a 1-unit increase in MSE-CF was not associated with an increase in the nonintensive group (β = 0.0613, *P* = 0.58). There was a signficant interaction between the intervention and MSE-CF (β = 0.390, *P* = 0.038). This indicated that, in the intensive group, at higher levels of MSE-CF there was an increased association between MSE-CF and feeding green leafy vegetables in the last 24 h. The mediation coefficient was significant in the intensive group (β = 0.345, *P* = 0.010) and not in the nonintensive group (β = 0.0469, *P* = 0.58): for every 1-unit increase in MSE-CF in the intensive group, there was a 0.345 increase in the log odds of feeding green leafy vegetables in the last 24 h. The simultaneous mediation and potentiation are graphically depicted in **Figure 2**.

### Egg

#### Intervention effect

For on-time introduction of egg, in a fully adjusted model without MSE-CF we found a 0.585 (*P* = 0.037) increase in the log odds of on-time introduction of egg in the intensive group compared with the nonintensive group (results not shown). These log odds corresponded to an 11.2 percentage-point increase in the predicted probability for the on-time introduction of egg associated with the intensive group (78.1% predicted probability) compared with the nonintensive group (66.9% predicted probability).

#### Mediation effect

When we included MSE-CF in the full model, the β-coefficient for the intervention was only slightly attenuated. We found a 0.537 increase in the log odds of on-time introduction of egg in the intensive group compared with the nonintensive group (*P* = 0.038). Because the effect of the MSE-CF scale on egg was not significant (*P* = 0.53; results not shown), we did not examine any mediating or potentiating role of MSE-CF for the on-time introduction of egg.

In sum, we found that, although the intervention had impacts on both green leafy vegetables in the last 24 h and the on-time introduction of egg, MSE-CF mediated and potentiated only the intervention effects on green leafy vegetables. MSE-CF did not mediate the significant intervention effect on the on-time introduction of egg.

## Discussion

We sought to clarify the role of self-efficacy in an effective intervention to improve CF behaviors in a low-income setting and chose to examine maternal self-efficacy in the context of 2 distinct behaviors. We found that impacts of the A&T intervention were mediated through increased MSE-CF in the case of feeding green leafy vegetables in the last 24 h. Furthermore, mothers with higher MSE-CF gained the most from the intervention on this behavioral outcome. In contrast, although the A&T intervention was also effective in increasing on-time introduction of eggs to infants as an early animal-source food, the intervention did not work through increased MSE-CF to affect this behavior, nor did MSE-CF potentiate this effect. Our research thus shows that the role of MSE-CF differs between these 2 IYCF behaviors.

The finding that MSE-CF both mediated and potentiated the impact of the intervention on feeding green leafy vegetables in the last 24 h (as depicted in Figure 2) supports the theory of “moderated mediation” (41). The mediation effects of MSE-CF were potentiated by the level of MSE-CF, such that the mediation effects were stronger among those with higher MSE-CF. In the intensive group, having a higher MSE-CF corresponded to a higher predicted probability of feeding green leafy vegetables in the last 24 h. In the absence of potentiation, the lines would have been parallel. To our knowledge, the combined mediation and potentiation effects of self-efficacy have not been shown before this study.

Our divergent findings with regard to the 2 recommended behaviors highlight the complex determinants of IYCF behaviors and empirically strengthen qualitative insights from the intervention's process evaluation (31). To interpret the difference between the findings for green leafy vegetables and eggs, we draw on these findings and field observations conducted as part of the larger study. Eggs, which must often enter the household from the market, are relatively expensive in Bangladesh, ∼0.12 US$/egg at the time of this study, whereas in 2010, 18.5% of the population lived on <1.90 US$/d and 56.8% of the population lived on <3.10 US$/d using 2016 World Bank poverty estimates (42). Furthermore, our qualitative research and field observations indicated that eggs are rarely a preferred food for adults in Bangladesh. Eggs, therefore, may fall into the category of being a “special food” for a child, and because of cultural considerations, a caregiver generally must request that someone buy this “special food” in the market for the child. This requires that the caregiver is able to influence the household shoppers. These differing determinants of specific complementary feeding behaviors have important implications for frontline workers who are usually charged with delivering BCC that is intended to address a diversity of CF practices. Our findings suggest that intervention design efforts should likely delve deep into the determinants of specific individual practices, and assess the feasibility of shaping some behaviors via counseling alone.

Although the present study focused primarily on MSE-CF, certain structural or cultural factors known to affect CF practices should be acknowledged. In Bangladesh, elders and men usually do the food shopping for the household. At baseline, women's reported control over the purchase of food, clothes, and medicines was low, with only 3.6% of mothers stating that they purchase most of the food consumed by the family (34). Men reportedly managed the household finances and decided what to purchase (34). This role structure that reduces women's autonomy has significant implications for access to resources and what is fed to the child. Green leafy vegetables, on the other hand, are part of a typical family meal and are also relatively easy to grow. In the 2 A&T intervention groups combined, 45.1% of households reported consuming green leafy vegetables in the last 24 h, whereas only 23.3% reported consuming egg. Green leafy vegetables may be gathered from surrounding fields or from household gardens within a mother's access and control. As a result, she would not have to rely on other members of the household to provide these items to her child. Therefore, in addition to self-efficacy, structural and cultural factors such as women's agency, autonomy, and access to resources, all known to underlie healthy behaviors (17), may have influenced the CF practices examined here.

Another factor that could help to interpret these findings is the relation between maternal dietary diversity and child dietary diversity. Nguyen et al. (33) found that, in Bangladesh, mothers consume vitamin A–rich fruit and vegetables (including green leafy vegetables), and a mother's consumption of such foods is associated with her children's consumption of the same. Thus, the recommendation to feed green leafy vegetables has the advantage of being part of established consumption patterns. In contrast, egg consumption was much lower for both mothers and children, perhaps indicating limited access to eggs or a lack of preference for eggs in this setting (33).

Self-efficacy can be conceptualized at the level of a specific behavior (e.g., feeding a specific food in a particular way) or a domain of behaviors (CF). Our results suggest that measuring domain-specific self-efficacy is refined enough to allow for behavior-specific interpretation, given adequate contextual knowledge. Comparing the differences between caregivers’ responses to green leafy vegetables and eggs, we see why it is important to examine the associations of domain-specific self-efficacy at the level of a specific behavior. The lack of association of MSE-CF with the on-time introduction of egg can be explained by cultural and economic factors that limit a mother's agency to implement healthy behaviors (17). This outcome specifically points out the influence of household role structure and economic decision making associated with roles. This finding is consistent with Bandura's (28) supposition that “under forcible disincentives or imposed social and physical constraints, individuals are disinclined to act on their self-efficacy beliefs” (p. 10). Strong self-efficacy is no match for a reality of restricted agency or limited resources. Where men are the decision makers, or where households are constrained by resources to act on information or confidence, improving MSE-CF may be associated with limited improvement. Indeed, given the trade-off between purchasing “special foods” and other crucial household expenses in very poor households, purchasers may be unable to provide special foods even if they are convinced of the importance of providing them. Poverty may thus limit the impact of a well-designed and well-delivered IYCF BCC intervention (43).

In sum, these results underline the need to approach IYCF intervention design and evaluation with the consideration of key constructs and theories, such as self-efficacy, that influence caregiver behavior. In addition, interventions that are able to complement effective individual approaches with approaches that address social, environmental, and contextual determinants may be able to amplify their impacts (44, 45).

Some limitations of our data and analyses are worth mentioning. First, this study did not measure or model the mechanisms by which MSE-CF is increased, and we did not investigate which component of the intervention was responsible for the increase in MSE-CF in the intensive group. We simply note that the A&T intensive intervention directly influenced MSE-CF, which was part of the program theory of change (25, 26). Second, this study was conducted in the middle of the overall evaluation cycle. The effects of MSE-CF may become stronger with longer exposure to the intervention, or be attenuated as behaviors become more normative and accepted and require less individual agency. Third, both outcome measures are self-reported, and therefore may be subject to both social desirability bias and measurement (recall) error. In the context of this study, we were not able to assess the role of social desirability, but the final impact evaluation ascertained that, in the context of Bangladesh, social desirability bias was negligible (26). Finally, we note some challenges with measurement of domain-specific self-efficacy. Although our questions were extensively cognitively tested, the Cronbach's α coefficient of the MSE-CF scale was 0.60, on the lower end of acceptable scale properties, as a generally accepted range is 0.70–0.95 (29). This lower scale consistency may be attributed to the limited number of questions (4) included in the scale. Further work on this measure to refine and potentially expand the set of questions that comprise the scale or the scaling of responses might further improve internal consistency and would advance research in this area.

In conclusion, although challenging, our study indicates the utility of measuring and testing the role of domain-specific self-efficacy. Identifying critical variables, such as MSE-CF, along the pathway between intervention and outcomes can inform both intervention design and evaluation. Studying MSE-CF along the program impact pathway for this intervention improved our understanding of the mechanisms for behavior change in this setting and helped highlight that a specific behavioral determinant targeted by the intervention did indeed play a significant mediating and potentiating role, but only for one of the behaviors. Our findings indicate that MSE-CF may manifest differently for different behaviors and that, for some behaviors, other constraints are likely more salient. A multipronged approach to BCIs that fully considers both intrinsic and extrinsic challenges to supporting specific behaviors and foods could help strengthen the impact of such interventions.

## Supplementary Material

Supplemental data

## Notes

Alive & Thrive is funded by the Bill & Melinda Gates Foundation (OPP50838) and managed by FHI 360. NIH (5T32HD007331).

Supplemental Table 1 is available from the “Supplementary data” link in the online posting of the article and from the same link in the online table of contents at https://academic.oup.com/jn/.

**Abbreviations used:**
A&T: Alive & Thrive
BCC: behavior change communication
BCI: behavior change intervention
CF: complementary feeding
IYCF: infant and young child feeding
MSE-CF: maternal self-efficacy for complementary feeding
OLS: ordinary least-squares.
