# Supplementary material for: The Pathways from a Behavior Change Communication Intervention to Infant and Young Child Feeding in Bangladesh Are Mediated and Potentiated by Maternal Self-Efficacy
Source: J Nutr. 2018 Feb 27;148(2):259–66. doi: 10.1093/jn/nxx048 (PMC6299133; doi:10.1093/jn/nxx048)
Supplement: Supplemental data [file nxx048_supp.docx]

Supplemental Table 1: Baseline characteristics and descriptive statistics on dependent, independent, and control variables of children between 6 and 24 mo of age in the overall evaluation sample

| Intervention group | Intensive  (*n* = 303) | Nonintensive  (*n* = 303) |
| --- | --- | --- |
| Age of child, mo | 15.1 ± 5.43 | 14.6 ± 5.05 |
| Gender of child, % female | 47.2 | 50.5 |
| Mother’s age, y | 26.5 ± 6.11 | 26.7 ± 6.16 |
| Maternal education, y of schooling completed | 4.93 ± 3.67 | 4.36 ± 3.81 |
| SES, all assets score | 12.7 ± 3.66 | 11.2 ± 3.66 |
| Household food insecurity^1^, HFIAS score | 2.65 ± 5.10 | 3.01 ± 5.34 |
| Young children consuming green leafy vegetables in the last 24 h, % | 25.1 | 34.3 |
| Young children with on-time introduction of egg (6–8 mo), % | 63.3  (*n* = 237) | 64.2  (*n* = 240) |
| Age of introduction of egg, mo | 8.14 ± 2.60  (*n* = 237) | 8.00 ± 2.46  (*n* = 240) |
| Households consuming green leafy vegetables in the last 24 h, % | 36.0 | 49.8* |
| Households consuming egg in the last 24 h, % | 26.1 | 30.0 |
| Note: Values are expressed as mean ± SD or %  *n*’s are not displayed when missing values are < 5%  ^1^ The range of possible scores for HFIAS is 0–27, with lower scores reflecting better food security  Unadjusted P-values: *P < 0.05; **P < 0.01; ***P < 0.001; ****P < 0.0001 | | |
